# Topical gel of mesenchymal stem cells-conditioned medium under TNF-α precondition accelerates wound closure healing in full-thickness skin defect animal model

**DOI:** 10.25122/jml-2019-0103

**Published:** 2022-02

**Authors:** Agung Putra, Sugeng Ibrahim, Adi Muradi Muhar, Novalia Kuntardjo, Bayu Tirta Dirja, Zenitalia Pasongka, Insan Sosiawan Tunru

**Affiliations:** 1. Stem Cell and Cancer Research (SCCR), Faculty of Medicine, Sultan Agung Islamic University, Semarang, Indonesia; 2. Department of Postgraduate Biomedical Science, Faculty of Medicine, Sultan Agung Islamic University, Semarang, Indonesia; 3. Department of Pathological Anatomy, Faculty of Medicine, Sultan Agung Islamic University, Semarang, Indonesia; 4. Department of Molecular Biology and Biochemistry, Faculty of Medicine, Universitas Katolik Soegijapranata, Semarang, Indonesia; 5. Doctoral Program of Medical and Health Science, Faculty of Medicine, Diponegoro University, Semarang, Indonesia; 6. Department of Surgery, Faculty of Medicine, Universitas Sumatera Utara, Medan, Indonesia; 7. Student of Postgraduate Biomedical Science Program, Faculty of Medicine, Sultan Agung Islamic University, Semarang, Indonesia; 8. Department of Microbiology, Faculty of Medicine, Mataram University, Mataram, Indonesia; 9. Postgraduate Biomedical Science Program, Faculty of Medicine, Udayana University, Bukit Jimbaran, Indonesia; 10. Department of Pathological Anatomy, Faculty of Medicine, YARSI University, Jakarta, Indonesia

**Keywords:** MSCs, MSC-CM-T, wound healing, PDGF, Fibroblast, ANOVA – Analysis of Variance, APC – Allophycocyanin, BSA – Bovine Serum Albumin, CD – Cluster of Differentiation, DMEM – Dulbecco’s Modified Eagle Medium, ELISA – Enzyme-linked Immunosorbent Assay, ERK – extracellular signal-regulated kinases, FBS – Fetal Bovine Serum, FITC – fluorescein isothiocyanate, HE – Haematoxylin and Eosin, HLA-DR – Human Leukocyte Antigen-DR, ISCT – International Society of Cellular Therapy, LSD – Least Significant Difference, MAPK – mitogen-activated protein kinase, MSC-CM-T – Mesenchymal Stem Cells-Conditioned Medium, MSCs – Mesenchymal Stem Cells, PBS – Phosphate Buffer Saline, PDGF – Platelet-derived Growth Factor, PerCP – peridinin chlorophyll protein, SD – Standard Deviation, T1 – Treatment 1, T2 – Treatment 2, TNF-α – Tumor Necrosis Factor-α, UC – Umbilical Cord, VEGF – Vascular Endothelial Growth factor, Veh – Vehicle Control

## Abstract

Mesenchymal Stem Cells (MSCs) under TNF-α stimulation (MSC-CM-T) can release numerous trophic and survival molecules that have a promising prospect in wound healing acceleration. However, the effective levels of MSC-CM-T in topical gel preparation to accelerate wound healing should be further explored. The aim of this study was to investigate the effects of MSC-CM-T in topical gel preparation in accelerating optimal wound healing through analyzing PDGF levels, wound closure rate percentages, and fibroblast density appearances. Twenty-four male Wistar rats were performed a full-thickness excision. The group studies were randomly assigned into four subgroups: control gel, control medium, and two treatment groups: MSC-CM-T topical gel at doses of 100 μL and 200 μL (T1 and T2, respectively). Wound closure rates were measured by standard caliper, platelet-derived growth factor (PDGF) levels were analyzed using ELISA on days 3 and 6, whereas the fibroblast density appearances were determined using hematoxylin-eosin staining. This study found a significant increase in PDGF levels in all treatment groups on days 3 and 6. These findings were in line with the increase of wound closure rates in all treatment groups on day 6, in which the high dose of MSC-CM-T was more effective in initiating the increase of wound closure rate. We also found the fibroblast density appearances on day 6 in the T2 group. We conclude that the topical gel of MSC-CM-T is more effective in accelerating wound closure healing through increasing PDGF levels and wound closure percentages and fibroblast density appearances in the skin defect animal models.

## Introduction

Optimum wound healing is a complex process involving a combination of soluble mediators and growth factors along with epithelial cells, fibroblasts, and endogenous mesenchymal stem cells (MSCs) to induce completely cutaneous-subcutaneous formation [1]. MSCs studies have become very popular in restoring wound healing models because of their critical role in controlling inflammatory responses and enhancing injured tissue regeneration through the multi-lineage differentiation capability and the release of numerous trophic-survival molecules, known as paracrine mechanisms [2, 3]. However, there are limited efficacies in using the transplanted MSCs due to low MSC survival rates in vivo, poor engraftment to injured areas, and the complicated techniques in maintaining the MSC stability in a culture laboratory [4]. Hence, a new approach to overcome those gaps is needed, such as using MSCs paracrine concept by introducing the soluble molecules secreted by MSCs into a culture medium under certain stimulation, known as MSC-conditioned medium (CM) [5].

As the primary resources of secreted factors, MSCs are characterized by the expression of CD90, CD73, CD105 and lack of expression of CD14, CD45, CD34, CD11b, CD31, and HLA-DR. In addition, MSCs have the potential to differentiate into osteocytes, chondrocytes, and adipocytes [6], and immunomodulatory capability through modulating regulatory T cells (T-reg) to control inflammatory cells [7–9]. Specifically, MSCs under TNF-α stimulation produce a multitude of soluble factors into a medium culture where MSCs are cultured [10, 11] that are referred to as secretome mechanisms. These secreted molecules of MSC-CM contain microvesicles and exosomes, particularly platelet-derived growth factor (PDGF) and vascular endothelial growth factor (VEGF), and also several anti-inflammatory cytokines that are crucial in wound healing [12]. PDGF is one of several polypeptide cytokines released by MSCs to regulate the activation, growth, and differentiation of a diverse cell type, particularly fibroblast, in accelerating the wound healing processes. In addition, PDGF also acts as chemoattractant molecules of inflammatory cells in initiating the inflammation processes, proliferation activities, and remodeling processes of wound healing [13–15].

The use of MSC-CM has a distinct advantage of being applicable via local administration, and also, the type and number of molecules released by these MSCs can be precisely quantified. MSC-CM has a promising prospect to be produced as pharmaceuticals in accelerating wound healing [4]. A major issue of using MSC-CM is the low concentrations of growth factors and anti-inflammatory cytokines in CM for effective therapy [16]. Several strategies of using MSC-CM were designed to increase the release of the proper molecules for optimal wound healings such as PDGF, VEGF, and IL-10 with high levels. One potential resolution to this issue was by preconditioning MSCs with TNF-α to significantly increase several growth factors, including PDGF levels in MSC-CM [10]. However, the effective levels of MSC-CM from TNF-α incubation, known as MSC-CM-T in topical gel preparation to accelerate the optimum wound healing, remains unclear. Given the beneficial role of MSC-CM-T as therapeutic potentials, we investigated the topical gel containing MSC-CM-T in accelerating wound healing by analyzing PDGF levels, wound closure percentages, and fibroblast density appearance.

## Material and Methods

### MSCs isolation and culture

Umbilical cords (UCs) from 19-day pregnant rats were collected under general anaesthesia. Under aseptic condition, the cords were cut into smaller pieces and transferred into a T25 culture flask (Corning, Tewksbury, MA, USA) containing Dulbecco’s modified eagle medium (DMEM) (Sigma-Aldrich, Louis St, MO), supplemented with 10% fetal bovine serum (FBS) (Gibco™ Invitrogen, NY, USA), 1% penicillin (100 U/mL)/streptomycin (100 μg/mL) (Gibco™ Invitrogen, NY, USA) and 0,25% amphotericin B (Gibco™ Invitrogen, NY, USA). These flasks were incubated at 37° and 5% CO_2_. The medium was renewed every 3 days, and the cells were passaged after reaching 80% confluency. The MSCs-like at passage 5 were employed for the following experiments.

### Marker characterization

MSCs-like surface antigens were analyzed by flow cytometry analysis at the fifth passage. After trypsinized and pelleted, the cells were subsequently incubated in the dark using fluorescein isothiocyanate (FITC)-conjugated, allophycocyanin (APC)-conjugated, peridinin chlorophyll protein (PerCP)-Cy5.5- conjugated CD90, CD73, and CD105 for 30 minutes at room temperature. The cells were rinsed twice using PBS. The analyses were performed using a BD Accuri C6 Plus flow cytometer (BD Bioscience, San Jose, CA, USA), and post-acquisition analysis was calculated using BD Accuri C6 Plus software (BD Bioscience, San Jose, CA, USA).

### Differentiation analysis

The MSCs-like were cultured in a density of 1.5×10^4^ cells/well. The cells were grown in 24 well plates with standard medium containing DMEM (Sigma-Aldrich, Louis St, MO), supplemented with 10% FBS (Gibco™ Invitrogen, NY, USA), 1% penicillin (100 U/mL)/streptomycin (100 μg/mL) (Gibco™ Invitrogen, NY, USA) and 0,25% amphotericin B (Gibco™ Invitrogen, NY, USA) at 37°C and 5% CO_2_. After reaching 95% confluency, the standard medium was aspirated and replaced using osteogenic differentiation medium containing Mouse MesenCult™ Osteogenic Differentiation Basal Medium (Stem Cell Technologies, Singapore) with 20% Mouse MesenCult™ Osteogenic Differentiation 5X Supplement (Stem Cell Technologies, Singapore), 1% L-Glutamine (Gibco™ Invitrogen, NY, USA), 1% penicillin (100 U/mL)/streptomycin (100 μg/mL) and 0,25% amphotericin B. The differentiation medium was renewed every 3 days. After bone matrix formation occurred, calcium deposition was visualized using alizarin red staining (Sigma-Aldrich, Louis St, MO).

### CM and gel preparation

The MSCs (1×10^4^ cells/well) were supplemented using 10 ng/ml TNF-α recombinant (BioLegend, San Diego, CA) in a 24-well plate using DMEM serum-free (Sigma-Aldrich, Louis St, MO), and incubated at 37°C and 5% CO_2_. After 24 hours of incubation, the MSC-CM was collected by centrifugation at 1900 rpm in 10 minutes. The topical gel of MSC-CM-T for T1 and T2 groups were made using supplemented base gel combined with 100 μL and 200 μL MSC-CM-T, respectively. On the other hand, supplemented base gel combined with 200 μL DMEM serum-free and base gel were used only for control medium and control gel, respectively.

### Full-thickness skin defect animal model and MSC-CM-T treatment

Twenty-four male Wistar rats weighing 200 g were caged at a 22±2°C and 60% relative humidity, with a 12:12-hour light-dark cycle. The animals were randomly divided into 4 groups: control medium, control gel, T1, and T2 groups (n=6 each group). To establish the animal model of full-thickness skin defect model, rats were anesthetized, and the dorsal skin was shaved and cleaned with a tincture of iodine. One 6 mm circular full-thickness biopsy punch excision was performed for each rat. The T1 and T2 groups were treated topically using supplemented base gel combined with 100 μL and 200 μL MSC-CM-T. On the other hand, the control medium and control gel groups were treated topically using supplemented base gel combined with 200 μL DMEM serum-free and base gel only. These interventions were applied twice a day until day 6.

### Wound closure measurement

The wound closure measurements were employed on days 3 and 6. Wound areas were determined using a standard caliper. The percentage of wound closure was calculated using: ,where A_0_ is the original wound area after wound creation, and A_t_ is the area of the wound at the time of measurement on days 3 and 6.

### ELISA

ELISA analysis was done in-vitro and in-vivo. For in-vitro analysis, the level of PDGF released in MSC-CM-T and DMEM serum-free were measured using specific ELISA. On the other hand, for in-vivo analysis, the rat serum from each group on days 3 and 6 was collected, and the PDGF level measurements were analyzed using specific ELISA. The PDGF analysis using a specific ELISA kit was performed according to the manufacturer’s instructions (Fine Test, Wuhan, China). The colorimetric absorbances were analyzed at a 450 nm wavelength using a microplate reader of the experiment.

### Fibroblast density appearances

Wound tissues on day 6 were fixed and blocked by formalin and paraffin. Horizontal sections were taken from each paraffin block. Haematoxylin and eosin (HE) staining was performed after dissolving the paraffin substances by processing xylene for 1 min. Fibroblast density appearances were described by the pathologist.

### Statistical analysis

Values were expressed as mean±SD. Two and more group comparisons were analyzed by ANOVA, followed by post hoc Fisher’s LSD. A P value of <0.05 was considered significant. All analyses were performed with SPSS 23.0.

## Results

### Characterization and differentiation of MSCs

**Figure 1.**
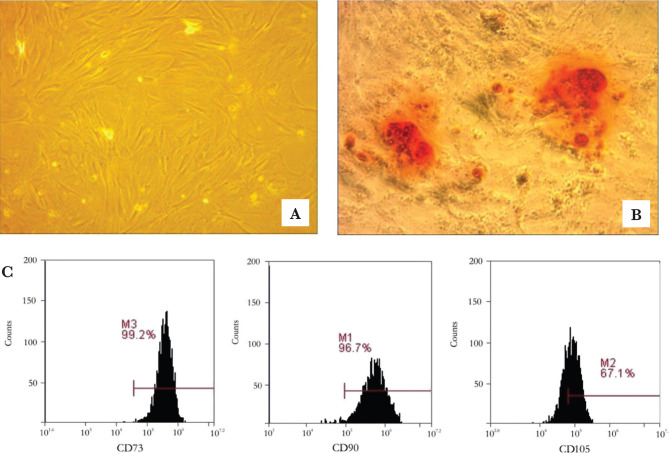
A – MSC-like from the in vitro culture showed fibroblast-like cells and polygonal shape, with 20x magnification; B – The osteogenic differentiation test with alizarin red staining appears red color in the MSCs population; C – The characterization analysis of UC-MSCs revealed that they could express a high level of CD73 (99.2±0.4%), CD90 (96.7±1.3%), CD105 (67.1±0.5%).

MSCs-like was isolated and cultured from umbilical cord based on their plastic adherent capability under a standard culture condition. In this study, the cell morphology of MSCs exhibited typical monolayers of spindle-shaped, fibroblast-like cells, with adherent capability to the plastic flask (Figure 1A). Isolated cells were cultured for 2-3 weeks in a monolayer and used for characterization and differentiation analysis after the fifth passage. At the end of the fifth passage’s expansion, osteogenic differentiation assay on MSCs-like was performed by administering the standard and osteogenic medium for 21 days. Calcium deposition was visualized in red color using alizarin red solution (Figure 1B). Moreover, the MSCs-like surface antigens were analyzed using flow cytometry. In this study, we found a high level of CD90 (96.7±1.3%), CD105 (67.1±0.5%) and CD73 (99.2±0.4%) (Figure 1C).

### Wound closure

**Figure 2.**
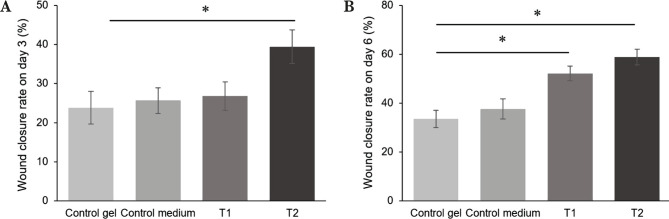
Wound closure rates were measured as the percentage of wound area on days 3 and 6 after the treatment of MSC-CM-T. A – There was a significant increase in wound closure rates in the T2 group on day 3. For Review Only; B – At day 6, we also found a significant increase in wound closure rates on all treatment groups.

After MSC-CM-T treatments in all groups, the length of the wound closure rates was measured in each group on days 3 and 6. The wound closure rates were calculated as a percentage number. The analysis of wound closure rates showed that there was a significant increase of the T2 group as a high dose of MSC-CM-T topical gel on day 3 (p<0.05) (Figure 2A). Interestingly, the wound closure rates were increased in time-dependent, in which we found a significant increase of wound closure rates in all treatment groups on day 6 (p<0.05) (Figure 2B).

### PDGF level

**Figure 3.**
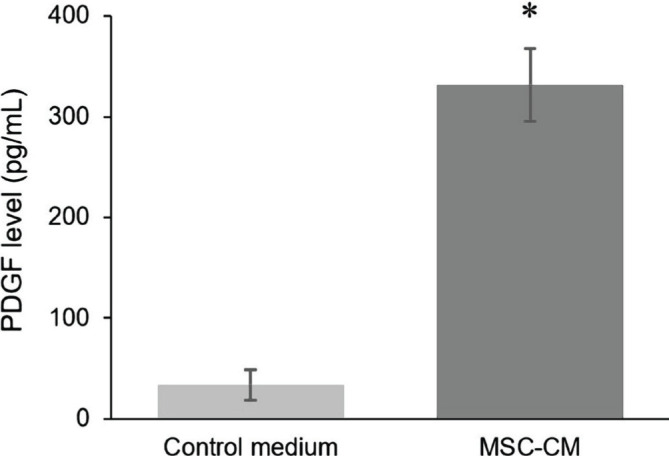
The level of PDGF on MSC-CM-T was significantly higher than the control medium, indicating the capacity of MSCs to express growth factors after TNF-α incubation.

**Figure 4.**
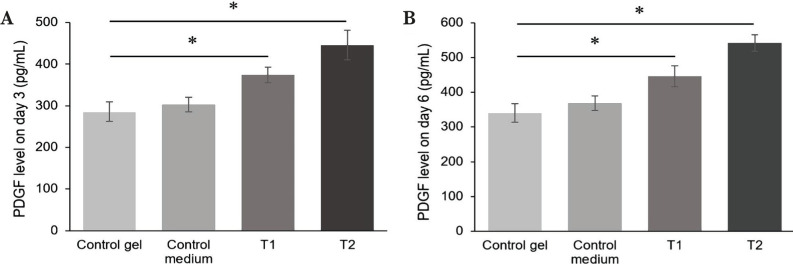
Comparison of the level of PDGF between groups on day 3 and day 6. The PDGF level was For Review Only significantly increased on T1 and T2 groups on day 3 A, and day 6 B.

Before being applied to the injury areas, the PDGF level in MSC-CM-T was analyzed using ELISA. We found that the PDGF level in MSC-CM-T was about 331.67 pg/ml, higher than the control medium (Figure 3). Furthermore, to explore the PDGF level in all treatment groups on days 3 and 6, we analyzed these molecules using ELISA. In this study, we found that the PDGF level significantly increased in all treatment groups on days 3 and 6 (p<0.05). The PDGF level of the T1 and T2 group on day 3 was about 374.158±18.120 pg/ml and 445.58±35.601 pg/ml (Figure 4A), while on day 6 was about 446.48±30.31 and 542.42±23.56, respectively (Figure 4B).

### Fibroblast density appearances

**Figure 5.**
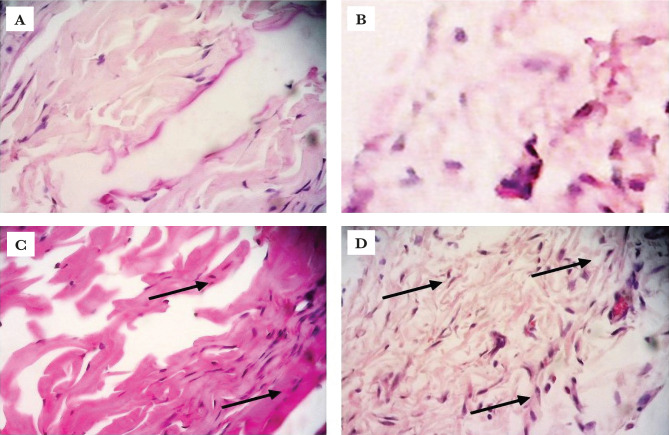
The H&E staining of fibroblast among all groups showed that there was fibroblast density appearance on treatment groups, as shown with arrows (A – control gel; B – control medium; C – T1 and D – T2).

To confirm the effects of MSC-CM-T on all treatment groups after day 6, we describe the fibroblast density appearances using Haematoxylin and Eosin (HE) staining. This study showed that the fibroblast density appearances were more visualized in all groups treated with MSC-CM-T. The appearance of fibroblast was shown by HE staining in Figure 5A–D.

## Discussion

Optimal wound healing requires a well-orchestrated integration of numerous cellular events mediated by several growth factors, cytokines, and chemokines [3]. Although MSCs have the homing capability into the sites of injury and release several trophic factors to accelerate wound healings [10, 11], the application of MSCs transplantation is still hindered by many obstacles, particularly the low engraftment efficiency, time-consuming procedures of preparation, the immune compatibility, tumorigenicity, embolism formations [17, 18]. Therefore, new promising approaches using the MSC-CM, soluble molecules released by MSCs under inflammatory conditions run across species barriers without the problems mentioned above.

**Figure 6.**
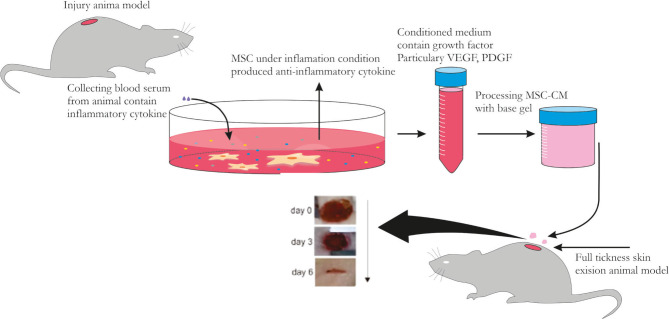
The mechanism of topical gel MSC-CM-T in promoting wound closure. Under TNF-α stimulation, MSCs could polarize into type 2 anti-inflammatory then release several cytokines such as PDGF to the medium (MSC-CM-T). The MSC-CM-T was collected and the topical gel of MSC-CM-T was made by combining supplemented base gel with the MSC-CM-T in various concentrations. The accumulation of PDGF from the gel in the injured area directly activates stromal and fibroblast cells to induce collagen formation leading to wound closure acceleration.

In this study, we used MSC-CM activated by 10 ng/ml TNF-α, known as MSC-CM-T, according to a previous protocol [10] in which a part of base gel combined with 5 and 10 parts of MSC-CM-T was used in treatment groups. We previously ran the analysis of PDGF levels in MSC-CM-T before applying to wound sites to precisely determine the level of PDGF contained in MSC-CM-T preparations. We found that PDGF levels in the MSC-CM-T preparation are 331.67 pg/ml, which are higher than MSC-CM preparations without TNF-α stimulation. This data indicated that the pre-activation of MSCs under inflammatory conditions is important to be performed before the CM collection. This is in accordance with previous studies that showed the MSC-CM-T contains several cytokines and growth factors, including PDGF [12]. The present study found that MSC-CM-T can restore the full-thickness skin defects by significantly increasing the PDGF level in all treatment groups on days 3 and 6. In addition, we also assumed that the PDGF in the MSC-CM-T topical gel plays a role as a chemoattractant of inflammatory cells in the initial phase of wound healing, then strongly promoting the skew of the inflammatory environment towards the proliferation phase to induce wound healing acceleration (Figure 6).

In line with the increase of PDGF, we also found a significant increase of wound closures in high doses of MSCs-CM-T groups (T2) on day 3; however, there was no significance in a low dose of MSCs-CM-T groups (T1). These data suggest that MSC-CM-T at high doses accelerates wound healing more than low doses. The increase of PDGF may initiate the binding of PDGF-to-PDGF receptor (PDGFR) around the injured cells, particularly the active fibroblast, triggering fibroblast activation, resulting in the ERK1/2 signal stimulation, the robust proliferation pathway [13]. The activated pathway of those signals implies the activation of mitogen-activated protein kinase (MAPK) [19], leading to the increase of c-fos expression associated with transcription of various growth factors, including PDGF [20].

The accumulation of PDGF following the MCS-CM-T administration in the injured area directly activates stromal cells, including fibroblasts that were in quiescent states previously. The activated fibroblasts produce massively collagen triggering cross-linking formation in the extracellular matrix (ECM) of wound closure devices [21]. On the other hand, the activated fibroblasts may also differentiate into the myofibroblast phenotype to strengthen the wound closure processes [22]. These data suggested that applying the high dose of MSC-CM-T in a topical gel is a promising preparation design in accelerating wound healing. However, in this study, we did not analyze the serial collagen deposit and VEGF level as a robust neovascular molecule, particularly in the remodelling phase; therefore, we do not know the prolonged effects of fibroblast activation after MSC-CM-T topical gen administration regarding fibrosis formations. We also did not analyze the remaining mesodermal differentiation of MSCs. The PDGF recombinant treatment results did not confirm that PDGF could be the major growth factor in mediating the wound healing process.

## Conclusion

This study confirmed our hypothesis of a great difference in the therapeutic efficacy of MSC-CM-T topical gel. The topical gel of MSC-CM-T is more effective in accelerating wound closure healing by increasing PDGF levels, wound closure percentages, and fibroblast density appearances in the skin defect animal models. Therefore, future work should focus on the potential clinical use of MSC-CM-T to optimize the quality, time, and delivery strategy into injury areas and further elucidate the MSC-CM-T mechanism.

## Acknowledgments

### Conflict of interest

The authors report no conflict of interest.

### Ethical approval

This study was approved by the Commission on Test Animal Ethics, Faculty of Medicine, Sultan Agung Islamic University (UNISSULA), Semarang, Indonesia (No. 204/VI/2017/Komisi Bioetik).

### Personal thanks

We want to thank the Stem Cell and Cancer Research Laboratory, Medical Faculty, Universitas Islam Sultan Agung Semarang for all facilities provided to finish this research.

### Authorship

AP contributed to conceptualizing, writing the original draft, and editing the manuscript. SI and AMM contributed to data curation. NK and ZP contributed to data collection. BTD contributed to methodology. ISAT contributed to data analysis.
